# The Molecular Mouse System: A New Useful Tool for Guiding Antimicrobial Therapy in Critically Ill Septic Patients

**DOI:** 10.3390/antibiotics13060517

**Published:** 2024-06-01

**Authors:** Carola Mauri, Alessandra Consonni, Elena Briozzo, Chiara Giubbi, Elisa Meroni, Silvia Tonolo, Francesco Luzzaro

**Affiliations:** Clinical Microbiology and Virology Unit, “A. Manzoni” Hospital, 23900 Lecco, Italy

**Keywords:** blood culture, antimicrobial resistance, Molecular Mouse, bloodstream infections, new antibiotics

## Abstract

Bloodstream infections (BSI) caused by multidrug-resistant (MDR) bacteria, pose a major threat for patients, especially for those who are immunosuppressed. Rapid pathogen detection and characterization from positive blood cultures are crucial in the management of patients with BSI to enable an adequate and timely antimicrobial therapy. This study aimed to investigate the potential role of the Molecular Mouse system, a new CE IVD molecular test designed to rapidly detect the causative agents of bacteremia and their resistance determinants, in the management of the therapy in critically ill patients. Agreement between the results of the Molecular Mouse and the conventional routine method was also considered. Overall, 100 positive blood cultures were collected from septic critically ill patients from May 2023 to January 2024 and analyzed with Molecular Mouse and routine protocols. The new instrument consistently agreed with the routine protocols in the case of monomicrobial blood cultures, while some discrepancies were obtained in the polymicrobial samples. Antimicrobial resistance genes were detected in 35 samples, with *vanA* and CTX-M-1/9 groups being the most frequently detected targets. Therapy was adjusted in 42 critically ill patients confirming the importance of new rapid molecular tests in the management of positive blood cultures, to adjust empirical therapy and use new antibiotics accurately.

## 1. Introduction

Bloodstream infection (BSI) is a major health burden worldwide. It is responsible for high mortality rates, worsened by the increasing spread of antimicrobial resistant pathogens, estimated to be responsible for 33,110 deaths in 2015 in Europe [1]. The occurrence of positive blood cultures in patients with systemic signs of infection, may be either primary or secondary to a documented source of infection and can lead to sepsis, organ failure, and death [2]. In the case of sepsis, timely detection and identification of the responsible pathogen is essential to provide appropriate antimicrobial therapy and to improve patients’ outcomes [3]. Moreover, the increasing prevalence of both Gram-positive and Gram-negative multidrug-resistant (MDR) organisms represents one of the greatest challenges to the effectiveness of BSI therapeutical treatments [4,5,6,7].

Traditional culture-based methods remain essential, but they are gradually being complemented by faster phenotypic techniques (e.g., identification and antimicrobial susceptibility testing performed directly from positive blood culture or after short sub-culture on solid medium) and/or rapid molecular methods [8]. In particular, new molecular approaches have been applied in the routine workflow to provide rapid identification and potential resistance profiles and to enable targeted therapeutic adequacy [9]. Extended characterization of resistance genes represents an additional value for appropriate therapy when considering that new commercially available antimicrobial compounds (including ceftolozane/tazobactam, ceftazidime/avibactam, meropenem/vaborbactam, imipenem/relebactam, cefiderocol, and, most recently, cefepime/taniborbactam) have specific antimicrobial activity against Gram-negative microorganisms depending on the produced enzyme(s).

Of importance, most of them include inhibitors that protect the associate drug (i.e., cephalosporin or carbapenem) from different types of carbapenemases (i.e., KPC, IMP, VIM, NDM, or OXA-48-like types). Ceftolozane/tazobactam is a new beta-lactam/beta-lactamase inhibitor combination with activity against some Gram-negative organisms, including extended-spectrum beta-lactamase (ESBL)-producing *Enterobacterales* and MDR and extensively drug-resistant *Pseudomonas aeruginosa*, but without action against carbapenemase-producing strains [10]. Another combination of a beta-lactam and beta-lactamase inhibitor is represented by ceftazidime/avibactam. This molecule is active against Ambler class A, class C, and some class D beta-lactamases, representing a first-line option against KPC- or OXA-48-producing *Enterobacterales* and an alternative against ESBL- or AmpC-producing *Enterobacterales* and *P. aeruginosa* [11]. Similarly, meropenem/vaborbactam is a beta-lactam/beta-lactamase inhibitor combination with action against beta-lactamases of class A (including ESBLs and KPCs) and C, but it is inactive against enzymes from class B and D [12]. Imipenem/cilastatin/relebactam is a combination of three molecules (carbapenem, cilastatin, and a beta-lactamase inhibitor) active against lactose-fermenting and non-fermenting Gram-negative bacteria, including strains producing ESBL, KPC, and AmpC, but not against those producing OXA-48. The molecules previously described are not active against *Acinetobacter baumannii* and metallo-beta-lactamases (MBL) producing Gram-negative organisms [12]. Otherwise, cefiderocol is a new antibiotic, a siderophore cephalosporin, active against a broad spectrum of Gram-negative pathogens, like ESBL and carbapenemase-producing strains (including KPCs, OXA-type serine beta-lactamases, and MBLs). Cefiderocol is composed of a drug–iron complex able to enter into the pathogen cell by both porins and the iron transporter system; it is unaffected by efflux pump overexpression. In this way, high periplasmic space concentrations are achieved [13]. The last beta-lactam/beta-lactamase inhibitor combination is represented by cefepime/taniborbactam. It is active against class A and class C beta-lactamases, as well as class B metallo-beta-lactamases, including VIM and NDM but not IMP-type enzymes. Taniborbactam acts as a reversible covalent inhibitor of serine beta-lactamases and as a competitive inhibitor of MBLs, restoring the activity of the antibiotic against many MDR and difficult-to-treat resistant (DTR) organisms [14,15]. Table 1 summarizes the microbiological targets of novel antimicrobial agents commercially available for Gram-negative organisms. Similarly, in the case of Gram-positive organisms, oritavancin and tedizolid are new antimicrobial agents with activity against *vanA* and *vanB* vancomycin-resistant enterococci (VRE) [16,17].

In this context, we evaluated the potential role of the Molecular Mouse system, in the management of the antimicrobial therapy of critically ill septic patients, especially in MDR strains. We also examined the agreement between the results provided by the molecular test and those obtained by the conventional routine microbiology method.

## 2. Results

From May 2023 to January 2024, 100 positive blood cultures obtained from critically ill septic patients were studied using the Molecular Mouse system. Of these, five samples were found to be negative using Molecular Mouse, because the target was not present in the panel (*Alkalihalobacillus clausii*, *n* = 1; *Bacteroides fragilis*, *n* = 1; *Parvimonas micra*, *n* = 1; *Pseudomonas oryzihabitans*, *n* = 1; *Sphingomonas paucimobilis*, *n* = 1), and these were excluded from the study. Therefore, a total of 95 samples were analyzed.

### 2.1. Bacterial Identification

Overall, 88 out of 95 bottles included in the study were monomicrobial samples, and 7 showed polymicrobial growth.

Among the monomicrobial samples (*n* = 88), the Molecular Mouse identified 78 microorganisms at the species level (Gram-negative, *n* = 59; Gram-positive, *n* = 19) and 10 at the genus level (Gram-positive, *n* = 7; Gram-negative, *n* = 3. The results obtained by the Molecular Mouse system fully agreed with those observed with routine culture protocol for monomicrobial samples by both rapid and conventional tests (Table 2). Concerning identifications at the genus level, the Molecular Mouse was not able to identify eight pathogens at species level because they were off-panel microorganisms (four *Streptococcus viridans group* and two *Streptococcus dysgalactiae equisimilis*, among the Gram-positive organisms; *Raoultella ornithinolytica* and *Providencia rettgeri* among the Gram-negative organisms). Otherwise, the Molecular Mouse system did not identify one *E. faecalis* and one *K. oxytoca*. In these two cases, there were amplifications curves of the corresponding microorganisms; however, they were beyond the detection limit of the instrument, which has a Ct value cut-off for a positive sample at 45. Amplification curves for identification at the genus level were detected.

Looking at the group of polymicrobial samples (*n* = 7), the results obtained by the Molecular Mouse method agreed with routine culture methods in five cases, while discrepancies were obtained in two samples (Table 3).

In particular, the Molecular Mouse identified 14 microorganisms (Gram-negative, *n* = 8; Gram-positive, *n* = 6) at the species level and one at the genus level (*Streptococcus* viridans group, not included in the panel). Concerning samples with discrepancies, the Molecular Mouse system was not able to identify one *E. faecalis*, one *P. aeruginosa*, one *Streptococcus* viridans group, and one *Streptococcus* viridans group that was not identified even at the genus level.

### 2.2. Resistance Genes

Antimicrobial resistance genes were detected in 35 samples (Table 4). Among the Gram negatives (detected in 69 samples), resistance genes were only found among *Enterobacterales* (24/58 samples), while no resistance genes were detected in the samples where *P. aeruginosa* (*n* = 7) and *A. baumannii* (*n* = 1) were identified. Only one carbapenemase (OXA-48) was found among the *K. pneumoniae* isolates. The resistance to cephalosporins mediated by CTX-M genes was found in 17 strains. Other resistance determinants, including the CMY-2 enzyme, were detected in single cases.

In the Gram-positive group (detected in 33 samples), resistance to vancomycin in the *E. faecium* strains was consistently carried by the *vanA* gene. Other *van* genes (*vanB*, *vanC1*, *vanC2/3*) were not detected in any sample.

### 2.3. Antimicrobial Therapy

Regarding the therapeutic impact, the Molecular Mouse results were evaluated in consideration of the ongoing empirical treatment, communicated by clinicians. The new rapid molecular system turned out to be decisive in 58/95 (61.1%) patients, thus determining a substantial change compared to an inadequate antimicrobial therapy or providing useful information for setting targeted therapy. In 26/58 (44.8%) cases, the therapeutic change was determined by the detection of resistance genes. The impact of the Molecular Mouse results on the therapies are shown in relation to the empirical treatment (Table 5) and microorganism identified (Table 6). Of note, in groups of organisms such as *Enterobacterales* and enterococci, where a high number of resistance genes were detected, changes in therapy were primarily due to the detection of those targets (Table 6).

## 3. Discussion

Antimicrobial resistance (AMR) is a major public health problem with a strong clinical and economic impact. In recent decades, it has assumed such global importance that it has led the World Health Organization and the European Union to adopt coordinated strategies and actions aimed to contain the phenomenon. The AMR situation in bacterial species, reported in the AMR surveillance networks, varies widely depending on species, antimicrobial group, and geographical region [18]. For years, Italy has been among the countries in Europe with the highest percentages of resistance to the main classes of antibiotics used in hospitals. In Italy, major matters of AMR are represented by resistance to third-generation cephalosporins in *K. pneumoniae* (53.3%) and *E. coli* (24.2%) and resistance to carbapenems in *Acinetobacter* species (88.5%) and *K. pneumoniae* (24.9%). Moreover, the significantly increasing trend in the percentage of vancomycin-resistant isolates of *E. faecium* further rose from 18.9% in 2018 to 30.7% in 2022 [19,20].

Several studies have already demonstrated the difficulties related to the treatment of MDR organisms responsible for BSIs and their association with high mortality rates. Among Gram-positive bacteria, the isolation of VRE is considered as an independent predictor of mortality in patients with enterococcal BSI [21], compared to infections caused by vancomycin-susceptible enterococci, and a delayed appropriate antibiotic therapy is considered as a determinant of an unfavorable outcome [22]. Therefore, the rapid identification of *Enterococcus* species and the presence of acquired vancomycin-resistant genes significantly reduce the time necessary to set up an appropriate therapy, decreasing the mortality rate [5]. Several studies described the association of infections from MDR Gram-negative organisms with unfavorable outcome and increased hospital stay [7,23,24,25,26,27,28,29]. In addition, time-appropriate antibiotic therapy has been shown to be an independent predictor of 30-day mortality in patients with KPC-producing *K. pneumoniae* BSI, and the onset of appropriate antibiotic therapy is recommended within 24 h following blood cultures’ collection [24].

The characterization of resistance genes is pivotal information for the clinical use of new antimicrobial compounds, especially in Gram-negative microorganisms. Since 2018, every year, the Italian National survey (AR-ISS) has released a report about the types of carbapenemases detected in *K. pneumoniae* and *E. coli* from BSIs (Table 7). These data are very useful to know the trend of diffusion of carbapenemases in these organisms, but they are not enough when evaluating the single patient.

In this scenario, rapid molecular assays represent a standard of care in the management of BSI. The Molecular Mouse is a recent CE IVD developed system that can identify a wide spectrum of microorganisms (including both Gram-positive and Gram-negative) as well as several determinants of resistance. In particular, among carbapenemases, the Molecular Mouse system encompasses KPC-type enzymes, as well metallo-beta-lactamase (IMP-, VIM-, and NDM-type enzymes) and oxacillinases (not only OXA-48-type but also OXA-23-type). From a therapeutical point of view, this activity is crucial since ceftazidime–avibactam is potentially active against KPC and OXA-48-type enzymes, whereas meropenem–vaborbactam and imipenem–relebactam are active against KPC enzymes only. None of these combinations acts against the metallo-beta-lactamases and oxacillinases typically produced by *Acinetobacter baumannii* (i.e., OXA-23 carbapenemases). In this context, an additional value of the Molecular Mouse is the capability of the system to detect the OXA-23 enzyme. Indeed, a progressive change from the blaOXA-58 to blaOXA-23 gene carriage was observed in Italy, with *A. baumannii* strains producing OXA-23 appearing in 2007 [35], and, as reported in a national Italian survey, carbapenem resistance in *A. baumannii* is primarily associated with the OXA-23 enzyme, which is the most commonly acquired carbapenemase [36]. On the contrary, OXA-58 oxacillinases, previously reported to be prevalent in Italy, were detected in few centers, even though they were distributed across the country [37,38,39]. These data also agree with the described worldwide dissemination of the blaOXA-23-like genes [40]. Other peculiarities of the Molecular Mouse system include detection of chromosomal genes determining low-level vancomycin resistance (vanC1 and vanC2/3) in *Enterococcus* spp. and targets for both CTX-M-1/9 groups and CTX-M-2/8 groups, SHV-type ESBLs, and the CMY-2 determinant, thus also allowing a better knowledge of local epidemiology.

The main advantages of molecular systems are certainly related to the reduction in the time necessary for the identification of microorganisms and eventually the presence of resistance genes; information regarding the identification and resistance mechanisms are available, respectively, at least 3 h and 24 h before the culture method results. In particular, the three cartridges tested on the Molecular Mouse system demonstrated good analytical performance as compared to the classic culture method. Moreover, the main advantage of the Molecular Mouse system is related to the modular configuration of the test; up to six analyses can be managed with single software. One sample can be tested simultaneously on different cartridges, and samples from different patients can be tested together. Moreover, the possibility to choose the chips to be tested based on microscopical examination certainly reduces the costs of the analysis. Beyond the demonstration of the analytical performance of the method, the main strength of this study is the analysis of the impact of the result of the Molecular Mouse testing in the clinical practice in the setting of critically ill patients (e.g., patients from intensive care units, cancer patients). The rapid identification of microorganisms and, eventually, resistance genes in positive blood cultures were demonstrated to have an important impact on the set up of appropriate therapy in more than 60% of cases.

On the other hand, the present study has some limitations. As reported, we investigated the analytical performance of three cartridges available for the Molecular Mouse platform. Therefore, we do not have any information on the performance of the instrument in the detection of Staphylococci and yeasts. The choice to test the chips for Gram-negative bacteria identification, Gram-negative bacteria resistance, and Gram-positive bacteria, excluding staphylococci identification, was guided by the fact that, in Italy, major concerns of antimicrobial resistance are related to Gram-negative bacteria with resistance to third generation cephalosporines and carbapenems and vancomycin-resistant enterococci. Another limitation of the study may be due to the selection of 100 positive blood cultures coming from critically ill patients. The microorganisms identified are not totally representative of the epidemiology of all positive blood cultures in our hospital; however, on the other hand critically ill patients are those who benefit the most from rapid choice or adjustment of the therapy related to the molecular detection of specific pathogens and mechanisms of resistance.

In conclusion, our data demonstrate that the Molecular Mouse system has the potential for early and accurate diagnosis of the causative pathogens of BSI. In particular, the molecular detection of resistance genes can rapidly improve the contribution for the selection of appropriate antimicrobial therapy, in little more than one hour from the positivity of the blood culture sample. Further studies could evaluate other cartridges of the instrument and the clinical outcomes.

## 4. Materials and Methods

### 4.1. Study Setting and Selection Criteria

This prospective study was performed from May 2023 to January 2024 at the ASST of Lecco (Italy), which consists of two hospitals, with a total of 950 beds. The following selection criteria were applied: positive blood culture samples were included in the study if they were collected from critically ill patients (e.g., patients from intensive care units, cancer patients); samples positive for Gram-positive bacilli or Gram-positive cocci in cluster at the Gram-stain and yeast were not investigated. Indeed, we decided to investigate samples positive for microorganisms that represent a major concern in antimicrobial resistance in Italy, such as Gram-negative bacteria and enterococci. A total of 100 positive blood cultures were studied using both the Molecular Mouse system (Alifax S.r.l., Polverara, Italy) and the conventional microbiology methods.

### 4.2. Blood Culture Diagnostics Following Conventional Culture Methods

Blood culture bottles (BacT/Alert Plus aerobic/anaerobic, bioMérieux, Marcy l’Etoile, France) were incubated in the BacT/AlertVirtuo^®^ instrument (bioMérieux, Marcy l’Etoile, France) at 37 °C for a maximum of 5 days (time for negative result). When a blood culture bottle was flagged as positive, it was removed by the instrument, and a Gram stain was performed. From all positive samples, the traditional workflow with overnight incubation was used. In particular, one drop of the blood culture broth was straked onto different agar plates and incubated at 36 °C for a maximum of 48 h in different atmospheres such as O_2_, 5% CO_2_, or an anaerobic atmosphere to enable bacterial growth. The agar media included Columbia agar with 5% sheep blood, chocolate agar with polyvitex, Columbia ANC agar with 5% sheep blood, MacConkey agar, and Schaedler agar with Vitamin K1 and 5% sheep blood.

If a monomicrobial bacterial population was detected by microscopic examination, a fast workflow was applied to these monomicrobial samples. The protocol is as follows: an aliquot (2.5 mL) of a monomicrobial sample was transferred to a tube with a gel separator (BD Vacutainer^®^ Blood Collection Tubes, Franklin Lakes, NJ, USA) and centrifuged at 3500 rpm for 10 min. The supernatant was discarded, and the pellet was inoculated on chocolate agar with polyvitex plate and incubated at 36 °C in 5% CO_2_ for 3 h.

In both cases, microbial identification was obtained by MALDI-TOF MS (Vitek MS, bioMérieux) after 3 h for the fast workflow or after overnight incubation for the traditional workflow. All colonies with different morphotypes were identified on plates after overnight growth. Similarly, from the point of view of timing, Antimicrobial Susceptibility Tests (ASTs) were performed according to the identified microorganism: VITEK 2 instrument (bioMérieux) for non-fastidious bacteria and the Kirby–Bauer diffusion susceptibility test according to the EUCAST Disk Diffusion Method guidelines for fastidious microorganisms [41,42]. If a multi-drug resistance microorganism was detected, antimicrobial susceptibility testing was performed also by the broth microdilution method (Sensititre, Thermo Fisher Scientific, Norristown, PA, USA). The results were interpreted according to the current EUCAST criteria, respectively, the 2023 and 2024 breakpoints [43,44].

**Figure 1:**
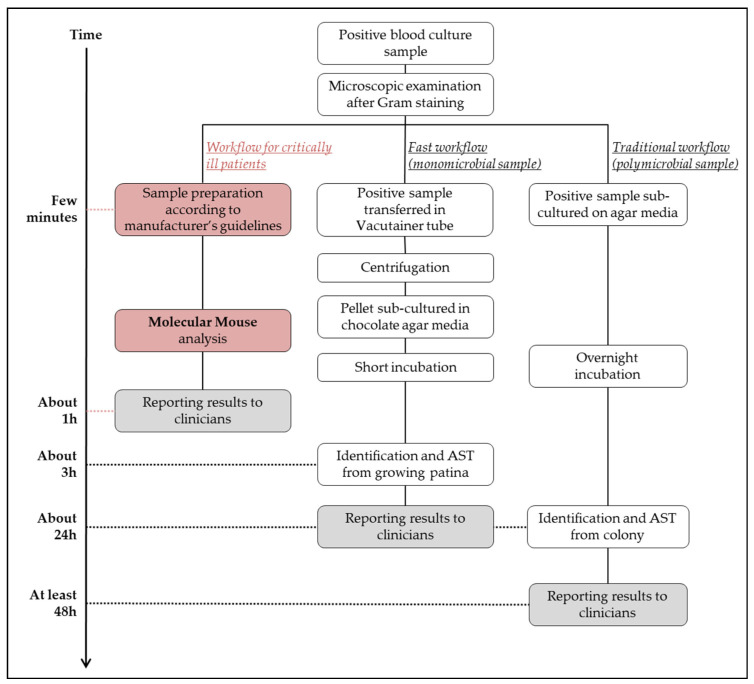
Summary of the blood culture diagnostics following the Molecular Mouse protocol (in red) and the conventional culture methods. The time necessary to report results to clinicians is also considered for the three different workflows.

A flowchart of these two conventional diagnostic methods and the Molecular Mouse analysis is represented in Figure 1.

### 4.3. Molecular Mouse System

The Molecular Mouse system allows the qualitative detection of DNA targets through real-time PCR by ready-to-use cartridges (or chips) with lyophilized reagents. The testing was performed according to the manufacturer’s instructions. Briefly, an aliquot (200 μL) of the positive blood culture was centrifugated for a first time to allow plasma/bacteria separation. Bacteria were then precipitated by a second step of centrifugation, resuspended in “DNAse free” water, and mixed with loading buffer. An aliquot (5 μL) of this mix was finally dispended into each dedicated cartridge well, where the necessary reagents were lyophilized. This was subsequently loaded into the instrument for molecular analysis using real-time PCR; no nucleic acid extraction is required (except for the yeast cartridge). In less than 60 min, this rapid molecular test can detect 65 targets: 44 for the identification of bacteria and fungi (Gram-negative bacteria, *n* = 15; Gram-positive bacteria, *n* = 20; yeast, *n* = 9), 21 for the detection of antimicrobial resistance genes (Gram-negative, *n* = 13; Gram-positive, *n* = 8). The molecular targets were grouped into different cartridges as reported in Table 8. A preliminary microscopic examination of the positive blood culture is required to choose the appropriate cartridge to be uploaded to the Molecular Mouse system. One software session can manage independently up to 6 instruments allowing testing of either one positive sample in more than one cartridge or different positive samples simultaneously.

In particular, in our work, we focused on three types of cartridges in relation to their impact in our epidemiological context: Gram-negative bacteria identification, Gram-negative bacteria resistance, and Gram-positive bacteria excluding staphylococci (identification and resistance are included in the same chip).

### 4.4. Impact of the Molecular Mouse System on the Setting of Antimicrobial Therapy

The results regarding the microorganism identification and resistance genes obtained by the Molecular Mouse system and the identification and phenotypic antimicrobial resistance profile by conventional culture methods were recorded in a standardized database. The reliability of the Molecular Mouse system was compared to conventional microbiology methods. Information about the antimicrobial therapy was also recorded. For each sample analyzed, the empirical antimicrobial therapy, as well as the intended choice of adjusted antimicrobial therapy after communication of the Molecular Mouse system result, were registered.

## Figures and Tables

**Table 1:** Microbiological targets of novel antimicrobial agents in Gram-negative organisms.

Antimicrobial Agent	Beta-Lactamase-Producing *Enterobacterales*				Non-Fermenting Gram-Negatives	
	ESBL	KPC	MBL	OXA-48	DTR-PA	CRAB
Ceftolozane/tazobactam	Yes	No	No	No	+/− ^a^	No
Ceftazidime/avibactam	Yes	Yes	No	Yes	+/− ^a^	No
Meropenem/vaborbactam	Yes	Yes	No	No	No	No
Imipenem/relebactam	Yes	Yes	No	No	+/− ^a^	No
Cefiderocol	Yes	Yes	Yes	Yes	Yes	Yes
Cefepime/taniborbactam	Yes	Yes	Yes	Yes	Yes	No

DTR-PA: difficult-to-treat *P. aeruginosa*; CRAB: carbapenem-resistant *A. baumannii*. ^a^: activity depends on the type of produced carbapenemase.

**Table 2:** Summary of the monomicrobial isolates detected by the Molecular Mouse system and then confirmed by routine culture methods. Isolates are grouped based on Gram staining results.

Gram-Negative (*n* = 62)	Gram-Positive, Excluding Staphylococci (*n* = 26)
*Escherichia coli/Shigella* spp. (*n* = 32) *Klebsiella pneumoniae* (*n* = 12) *Pseudomonas aeruginosa* (*n* = 7) *Enterobacteriaceae ^a^* (*n* = 3) *Haemophilus influenzae* (*n* = 2) *Klebsiella aerogenes* (*n* = 2) *Acinetobacter baumannii* (*n* = 1) *Klebsiella oxytoca* (*n* = 1) *Proteus mirabilis* (*n* = 1) *Serratia marcescens* (*n* = 1)	*Enterococcus faecium* (*n* = 11) *Streptococcus* spp. *^b^* (*n* = 6) *Streptococcus pneumoniae* (*n* = 3) *Enterococcus faecalis* (*n* = 2) *Streptococcus agalactiae* (*n* = 2) *Streptococcus pyogenes* (*n* = 1) *Enterococcus* spp. *^c^* (*n* = 1)

Gram-positive excluding staphylococci: Gram-positive bacteria with bacilli or streptococci or enterococci morphology at the Gram staining; *^a^*: *Raoultella ornithinolytica*, *Providencia rettgeri*, and *K. oxytoca* were identified as *Enterobacteriaceae*; *^b^*: *Streptococcus viridans* group (*n* = 4) and *Streptococcus dysgalactiae equisimilis* (*n* = 2) were identified at the genus level as *Streptococcus* spp.; *^c^*: *E. faecalis* was identified at the genus level as *Enterococcus* spp.

**Table 3:** Summary of the microorganisms identified by the Molecular Mouse system and the routine culture method in polymicrobial samples.

Molecular Mouse System	Routine Culture Method
*E. cloacae*, *E. faecium*	*E. cloacae*, *E. faecium*
*E. coli/Shigella* spp., *E. faecium*	*E. coli*, *E. faecium*
*E. coli/Shigella* spp., *E. faecium*	*E. coli*, *E. faecalis*, *E. faecium*, *Streptococcus* viridans group
*E. faecalis*, *K. oxytoca*	*E. faecalis*, *K. oxytoca*, *P. aeruginosa*
*E. faecalis*, *K. oxytoca*, *P. mirabilis*	*E. faecalis*, *K. oxytoca*, *P. mirabilis*
*E. faecium*, *K. oxytoca*	*E. faecium*, *K. oxytoca*
*P. aeruginosa*, *Streptococcus* spp.	*P. aeruginosa*, *Streptococcus* viridans group

Microorganisms revealed only by the routine culture method are underlined.

**Table 4:** Resistance genes by the Molecular Mouse system and the results from routine culture methods.

Molecular Mouse System	Routine Culture Method
*E. faecium*, *vanA* (*n* = 11)	Vancomycin-resistant *E. faecium*
*E. coli/Shigella* spp. CTX-M-1/9 groups (*n* = 11)	ESBL-producing *E. coli*
*K. pneumoniae* SHV (*n* = 6)	Wild-type *K. pneumoniae*
*K. pneumoniae* CTX-M-1/9 groups, SHV (*n* = 5)	ESBL-producing *K. pneumoniae*
*E. coli/Shigella* spp. CTX-M-1/9 groups, CMY-2 (*n* = 1)	ESBL-producing *E. coli*
*K. pneumoniae* CTX-M-1/9 groups, SHV, OXA-48 (*n* = 1)	Carbapenem-resistant *K. pneumoniae* (OXA-48 enzyme)

**Table 5:** Summary of the empirical therapy and adequacy in relation to the Molecular Mouse results.

Empirical Therapy	Total	No Therapy Change	Therapy Set or Adjusted ^*b*^
β-lactam agent + inhibitor	28	15	13
Antimicrobial combination therapy	24	9	15
Therapeutic window *^a^*	15	0	15
Third generation cephalosporin	13	6	7
Carbapenem (meropenem)	7	5	2
Glycopeptide (vancomycin)	4	2	2
Fluoroquinolone (ciprofloxacin)	2	0	2
Aminoglycoside (gentamicin)	1	0	1
Lincosamide (clindamycin)	1	0	1
Total	95	37	58

*^a^*: 48 h of washout before taking new antimicrobials; *^b^*: therapy was set or adjusted in relation to the Molecular Mouse results.

**Table 6:** Impact of the Molecular Mouse system results on the therapeutic choice.

Molecular Mouse Result	No Therapy Change	Set Up Targeted Therapy ^*a*^	Therapy Changed or Optimized	
			Organism-Based	Gene-Based
*Enterobacterales* (*n* = 52)	24	8	5	15
Enterococci (*n* = 14)	3	1	1	9
Streptococci (*n* = 12)	5	5	2	0
*P. aeruginosa* (*n* = 7)	3	1	3	0
*H. influenzae* (*n* = 2)	1	1	0	0
*A. baumannii* (*n* = 1)	0	0	1	0
Polymicrobial samples (*n* = 7)	1	0	4	2
Total (*n* = 95)	37	16	16	26

*^a^*: therapy was set up in relation to the Molecular Mouse results.

**Table 7:** Types of carbapenemases detected in *K. pneumoniae* and *E. coli* from BSIs in Italy from 2018 to 2022 [30,31,32,33,34].

Carbapenemases	2018	2019	2020	2021	2022
**KPC**	93.1%	85.9%	83.7%	80.1%	82.5%
**OXA-48**	2.2%	1.3%	1.8%	2.2%	4.6%
**MBL**	3.4%	11.4%	7.4%	13.1%	8.4%
**NDM**	na	na	4.8%	10.6%	7.2%
**IMP**	na	na	na	1.9%	0.8%
**VIM**	na	na	2.0%	0.1%	0.1%
**Double carbapenemases**	0.6%	0.5%	4.4%	3.2%	2.6%
**Not interpretable**	0.6%	1.0%	2.7%	1.4%	1.9%

na: data not available; not interpretable: discrepancy between the genotypic and phenotypic results.

**Table 8:** Summary of the targets available for testing on the Molecular Mouse system.

Gram-Negative Bacteria Identification	Gram-Negative Bacteria Resistance	Gram-Positive Bacteria, Excluding Staphylococci	Gram-Positive Bacteria Staphylococci	Yeast
*Acinetobacter baumannii* *Enterobacteriaceae* *Klebsiella aerogenes* *Enterobacter cloacae* *Escherichia coli/Shigella* spp. *Haemophilus influenzae* *Klebsiella oxytoca* *Klebsiella pneumoniae* *Neisseria meningitidis* *Proteus* spp. *Proteus mirabilis* *Pseudomonas aeruginosa* *Salmonella typhi* *Serratia marcescens* *Stenotrophomonas maltophilia*	KPC VIM NDM IMP OXA-23-like OXA-48-like SHV SHV ESBL CTX-M-1/9 groups CTX-M-2/8 groups CMY-2 mcr-1 mcr-2	*Bacillus subtilis* *Enterococcus* spp. *Enterococcus faecalis* *Enterococcus faecium* *Listeria monocytogenes* *Streptococcus* spp. *Streptococcus agalactiae* *Streptococcus anginosus* *Streptococcus pneumoniae* *Streptococcus pyogenes* *vanA* *vanB* *vanC1* *vanC2/3*	*Staphylococcus* spp. *Staphylococcus aureus* *Staphylococcus epidermidis* *Staphylococcus haemolyticus* *Staphylococcus lugdunensis* *Staphylococcus sciuri* *Staphylococcus hominis* *Staphylococcus simulans* *Staphylococcus saprophyticus* *Staphylococcus xylosus* *mecA* *mecC* *SCCmec-orfX* *vanA* and *vanB*	*Candida albicans* *Candida glabrata* *Candida krusei* *Candida parapsilosis* *Candida tropicalis* *Candida auris* *Candida lusitaniae* *Candida dubliniensis* *Candida guilliermondii*

## Data Availability

All data relevant to the study are included in the article.
